# Obsessive–Compulsive Tendencies Are Related to a Maximization Strategy in Making Decisions

**DOI:** 10.3389/fpsyg.2018.00778

**Published:** 2018-05-22

**Authors:** Ela Oren, Reuven Dar, Nira Liberman

**Affiliations:** School of Psychological Sciences, Tel Aviv University, Tel Aviv, Israel

**Keywords:** obsessive compulsive disorder, OCD, maximization, decision making, indecisiveness

## Abstract

The present studies were motivated by the hypothesis that attenuated access to internal states in obsessive-compulsive (OC) individuals, which leads to extensive reliance on external proxies, may manifest in a maximizing decision making style, i.e., to seeking the best option through an exhaustive search of all existing alternatives. Following previous research, we aimed to explore the possible relationships between OC tendencies, seeking proxies for internal states, indecisiveness and maximization. In Study 1, we measured levels of OC tendencies, seeking proxies for internal states, indecisiveness, maximization, depression and anxiety in an online Hebrew speaking sample (*N* = 201). In Study 2, we administrated the same questionnaires to an online English speaking sample (*N* = 240) and in addition, examined participants’ decision making strategies in a hypothetical situation. The participants in both studies were unscreened adults. Correlational and linear regressions analyses indicated that OC tendencies are related to maximization, even when levels of indecisiveness, depression and anxiety are controlled for. Moreover, the findings suggested that reliance on external proxies may partially account for the aforementioned association. Possible implications and future directions are discussed.

## Introduction

Obsessive compulsive disorder (OCD) is defined by two main components: obsessions and compulsions (American Psychiatric Association, 2013). The clinical phenomenology of OCD, however, is much richer, and includes, among other symptoms, a marked difficulty in making decisions. In fact, past and future decisions are often the content of obsessions for obsessive-compulsive (OC) individuals (i.e., individuals with clinical or subclinical OCD). For example, OC individuals might experience difficulty committing to a romantic partner because they are unsure whether that is the right decision. They may obsessively search the internet for a robot vacuum cleaner, feeling compelled to compare more and more brands on more and more attributes, or find themselves paralyzed in a supermarket aisle, trying to choose between two brands of cat food.

Much theorizing and research addressed constructs that are closely related to decision making in OCD, such as difficulty in achieving a sense of completion (Summerfeldt, 2004, 2007), “not just right experiences” (Coles et al., 2003) or a “feeling of knowing” (Szechtman and Woody, 2004; Boyer and Liénard, 2006). This difficulty is closely associated to the concept of doubt: without a proper sense of completion or a “feeling of knowing,” one would doubt just about anything – from “Did I lock the door?” to “Is s∖ he the right partner for me?” Nevertheless, surprisingly little research attempted to use theories of decision making to characterize the difficulty that people with OCD might have in making decisions (but see Frost and Shows, 1992, which we discuss later).

The present studies attempted to close some of this empirical and theoretical gap. Specifically, based on the Seeking Proxies for Internal States model of OCD (SPIS; Liberman and Dar, 2009; Lazarov et al., 2010), we proposed that OC tendencies would be characterized by a decision making style known as maximization (Schwartz, 2004). In what follows, we first describe maximization and the problem it poses to decision makers. We then describe the SPIS model of OCD and its relevance to difficulty in making decisions in general and to maximization in particular. We also distinguish maximization from indecisiveness, a construct that has been studied in relation to OC tendencies by Frost and Shows (1992). Finally, we describe our hypotheses in more concrete operational terms and test them in two studies.

In his seminal work “Rational choice and the structure of the environment” Simon (1956) pointed out that although rational choice theory postulates that individuals should attempt to consider as many alternatives as possible in order to optimize (i.e., to maximize the expected value of) their decisions, this behavior cannot in fact be adopted by agents who are bound by time and capacity constraints. As a result, in real life people would usually settle for a satisfactory, imperfect alternative, despite being aware that a better alternative might in principle exist.

Building on this earlier work, Schwartz (2000, see also Schwartz et al., 2002) proposed that only some individuals (the “satisficers”) manage to effectively settle for a satisficing decision alternatives, whereas others continue in an endless and (as a result necessarily) futile search for better alternatives. Schwartz and his colleagues coined the term “maximizers” to describe the latter style of decision making. According to their theory, maximizers seek and prefer unconstrained choice and always aim for the best, whereas satisficers seek and prefer situations in which the choice is limited and aim for options that are “good enough.” Schwartz (2000) theorized that a tendency to maximize would be associated with dissatisfaction, tension and regret. This is because it is usually difficult and even impossible to accrue enough information about all the options in order to make a choice. With more options, the standards for an acceptable outcome tend to increase. Moreover, when many options are available, people might end up believing that any undesired result is their own fault, because they should have been able to find a suitable option out of the many options available. For the satisficer, on the other hand, adding options after a decision has been reached does not induce regret; new options are often simply ignored. In support of this theorizing, it has been found that maximizers are particularly inclined to suffer from depression (Schwartz et al., 2002) and anxiety (Schwartz et al., 2002; Schwartz, 2004).

The *Seeking Proxies for Internal States* (SPIS; Liberman and Dar, 2009; Lazarov et al., 2010) model postulates that a core feature of OCD is impaired access to internal states, which drives OC individuals to seek and use more easily discernable indices, or “*proxies*,” for those states. In the SPIS model, internal states include cognitive functions, emotions and preferences, as well as bodily states and sensations. For example, an OC individual might find it difficult to access his/her feeling of hunger and cravings for specific foods, and therefore might resort to various objective considerations to decide what and when to eat (e.g., by checking how long it’s been since his/her last meal and applying rigid rules about the optimal ratio of proteins to carbohydrates in a single meal).

Several experimental studies have provided support for the SPIS model. Most of these have used biofeedback as proxy for the internal states of relaxation and muscle tension. These studies found that reliance on both genuine and false feedback was related to OC tendencies and was more evident in participants with high OC tendencies compared to participants with low OC tendencies (Lazarov et al., 2010, 2012a,b), and even more so in participants with OCD compared to both non-clinical and anxiety disorder participants (Lazarov et al., 2014). A more recent series of studies by Dar et al. (2016) suggested that OC tendencies are related to attenuated access to emotions. Specifically, OC tendencies were related to lower scores on the Experiential area of the Mayer-Salovey-Caruso Emotional Intelligence Test (MSCEIT; Mayer et al., 2002; Palmer et al., 2005), which relies on access to experienced emotions, but not on the Strategic area, which relies on semantic knowledge about emotions.

Moving beyond experimental laboratory procedures into the realm of daily experiences, Liberman and Dar (unpublished) recently developed an inventory for assessing reliance on external proxies for diverse internal states including hunger, enjoyment, interpersonal liking and understanding texts. The proxies for those states included other people’s opinions, the person’s own behavior, objective indices like the time elapsed since eating and *a priori* fixed rules. Scores on this inventory positively correlated with OC tendencies, even after controlling for concurrent depression and anxiety.

There are two ways in which the SPIS model can be related to the distinction between satisficing and maximizing in decision making (Simon, 1956; Schwartz, 2000). First, being satisfied with a decision alternative is an internal state with no clear, verifiable criterion. Individuals with OC tendencies might find it difficult, or even impossible, to access and rely on satisfaction with any available alternative. Second, stopping an action that has no clear end-state often relies on feeling satisfied with what one did so far (e.g., “I washed my hands enough and therefore I can stop”). This sense of satisfaction with what one has done might also be difficult to access for people with high OC tendencies. Indeed, one of the prevalent symptoms of OCD is the difficulty to stop specific actions that do not have a clear end-state, such as checking and washing (for a closely related theory of repeated action in OCD see Summerfeldt’ (2004, 2007) concept of incompleteness in OCD). Information search in decision making might be just another example of action with no clear stopping point, which people typically stop when they feel that they have done enough. People high in OC tendencies might find it difficult to access and/or to rely on this sense of satisfaction with their decision process, and thus would continue searching for additional decision-relevant information. Because of the difficulty to access and/or to rely on their own feeling of satisfaction with either the choice alternatives or the decision process, people high in OC tendencies would be drawn to maximization, which involves continuous search and an extensive reliance on proxies in the form of objective criteria and others people’s choices, opinions, reviews and ratings.

As mentioned earlier, research on decision making styles in OCD is scarce. A notable exception is a study by Frost and Shows (1992), which demonstrated increased indecisiveness in OC individuals. To measure indecisiveness, the authors constructed a 15-item scale in which participants indicated the extent to which different statements described them (e.g., *“I try to put off making decisions,” “I like to be in a position to make decisions (reversed),” “when ordering from a menu, I usually find it difficult to decide what to get”*). Using this scale, the authors found that OC tendencies were associated with increased levels of indecisiveness (for an experimental demonstration of indecisiveness with respect to perceptual decisions in people high in OC tendencies, see also Sarig et al., 2012).

The tendency to maximize is merely one of several possible reasons for indecisiveness. There are of course other reasons why reaching a decision might be difficult. For example, one could procrastinate, or fail to decide between only two available options (i.e., when maximization is irrelevant). Theoretically, then, maximization should have been one of the components of the indecisiveness scale. Empirically, however, the items in Frost and Shows’ self-report scale do not reflect maximization, and as a consequence the specific relation between OC tendencies and maximization has not been examined in their research. For instance, trying to put off making decisions (one of the items in Frost and Show’s scale) might reflect procrastination rather than an attempt to maximize. This implies that the relation between OC tendencies and maximization, which is the focus of our studies, should be independent of the relation between OC tendencies and indecisiveness, as measured by the scale developed and used by Frost and Shows (1992).

In the two present studies we aimed to explore the hypothesized positive relationships between OC tendencies and maximizing. We expected that maximizing would be uniquely related to OC tendencies, over and above any relation that OC tendencies might have with indecisiveness (as demonstrated by Frost and Shows, 1992). We also predicted the relation between OC tendencies and maximizing to hold even when controlling for depression and anxiety, which tend to be associated to OC tendencies (see Bartz and Hollander, 2006) and which also were found to be related to maximization. Finally, we expected that the variance that would be shared between maximization and OC tendencies would also be shared between maximization and the tendency to use proxies for internal states.

## Materials and Methods

### Study 1

#### Participants

Two hundred and one Hebrew speaking participants were recruited for the study by the “Midgam,” an online portal for recruiting survey participants. The sample was a fairly representative sample of the Israeli Jewish population. The study was approved by the Tel Aviv University ethics committee. Participants signed informed consent forms online and received a small monetary payment for their participation immediately after completing the study. The sample consisted of 53% men and had a mean age of 39.0 years (*SD* = 13.15; range: 18–64). Participants’ mean years of formal education was 14.29 (*SD* = 2.53; range: 9–23). All participants were recruited and completed the study in January 2016.

#### Measures and Procedure

##### Obsessive–Compulsive Inventory-Revised (OCI-R; Foa et al., 2002)

The OCI-R lists 18 characteristic symptoms of OCD. Each symptom is followed by a 5-point Likert scale ranging from 0 (not at all) to 4 (extremely), on which participants indicate the symptom’s prevalence during the last month. The OCI-R has been shown to have good validity, test–retest reliability and internal consistency in both clinical (Foa et al., 2002) and non-clinical samples (Hajack et al., 2004).

##### Seeking Proxies for Internal States Inventory (SPISI; Liberman and Dar, unpublished)

The SPISI lists 15 statements relating to one’s tendency to rely on external cues in order to deduce about internal states. For example: “*To know how hungry I am, I consider what and when I’ve eaten today.*” Each statement is followed by a 5-point Likert scale ranging from 1 (not at all) to 5 (extremely), on which participants indicate the extent to which the statement applies to them. The SPISI has been shown to have good validity, test–retest reliability and internal consistency (Liberman and Dar, unpublished).

##### Indecisiveness Scale (Frost and Shows, 1992)

To measure indecisiveness, Frost and Shows developed a 15-item Likert-type questionnaire (1992), based on an earlier investigation (Frost and Gross, 1993). Participants indicated the extent to which they agreed or disagreed with each item. Responses were made on a 5-point scale from “strongly disagree” to “strongly agree.” The internal reliability of this scale was good (Frost and Shows, 1992). The English version of the scale was translated to Hebrew using a back translation procedure.

##### Maximization Scale (Nenkov et al., 2008)

The 6-item maximization scale measures the tendency to maximize within decision making contexts. Ratings are made on a 5-point scale (1 = completely disagree, 5 = completely agree). Higher scores indicate a greater tendency to make decisions based on optimality versus sufficiency. Nenkov et al. (2008) concluded that compared to the original 13-item maximization scale and to the following 9-item maximization scale, the 6-item maximization scale performs best in terms of reliability and validity. The English version of the scale was translated to Hebrew using a back translation procedure. As one of the items deemed somewhat anachronistic (*“Renting videos is really difficult. I’m always struggling to pick the best one”*), we decided to exclude it from the scale and replaced it with a new item, more relevant to our research question (“*When I’m in a relationship, I’m bothered by the thought that there may be another wo∖ man, who could be better for me*”).

##### Depression, Anxiety and Stress Scales (DASS-21; Lovibond and Lovibond, 1995)

The DASS-21 is a self-report measure of 1-week state negative affect, with the specific aim of achieving maximal differentiation between the affective symptoms of depression, anxiety and stress. It comprises 21 items which determine the negative emotional symptoms experienced by the participant over the previous week, divided to three subscales: depression, anxiety and stress. Items are scored on a 4-point Likert-type scale from “did not apply to me at all” to “apply to me very much, or most of the time.” The factor structure of the DASS-21 is stable, and its scales possess good convergent and divergent validity and high internal consistency in clinical and in non-clinical samples and in different ethnic groups in adults (Lovibond and Lovibond, 1995; Antony et al., 1998; Lovibond, 1998; Henry and Crawford, 2005). For the purposes of the current studies, we used only the seven items related to depression and the seven items related to anxiety.

Participants completed all questionnaires in a fixed order online, using the “Midgam” project website^1^ (see **Appendix 1**).

### Study 2

The goals of Study 2 were to replicate Study 1’s findings with a different population (to allow the generalization of Study 1’s findings) and to further extend the findings by looking at the relation between OC tendencies and a difficulty in making a concrete real-life decision concerning a smartphone.

#### Participants

Two hundred and forty English speaking participants were recruited for the study by “Prolific,” an online portal for recruiting survey participants. The sample was a student sample from the United Kingdom. The study was approved by the Tel Aviv University ethics committee. Participants signed informed consent forms and received a small monetary payment for their participation immediately after completing the study. The sample consisted of 54% men and had a mean age of 25.77 years (*SD* = 4.72; range: 18–39). Participants’ mean years of formal education was 17.31 (*SD* = 3.06; range: 9–28). All participants were recruited and completed the study between July 2017 and August 2017.

#### Measures and Procedure

The measures used in Study 2 were similar to those used in Study 1. In addition, we included a set of questions about deciding on a smartphone. Specifically, participants imagined that their smartphone broke and they needed to choose a new smartphone. Nine questions gauged their intended extent of information search. For example: “You can read other users’ reviews of the specific brand you’re considering to buy. How many reviews would you like to read?” Participants could choose between (a) just one review, (b) 2–3 reviews, (c) 4–10 reviews, or (d) 11 reviews or more (see ‘the smartphone decision index’; **Appendix 2**). Participants completed all parts of the study in a fixed order online, using Prolific’s website^2^.

All procedures performed in the studies involving human participants were in accordance with the recommendations of the Tel-Aviv University research committee with written informed consent from all participants. All participants gave written informed consent in accordance with the Declaration of Helsinki. The protocol was approved by the Tel-Aviv University research committee.

In both Study 1 and Study 2, we hypothesized that:

(1) OCI-R scores would be uniquely related to maximization levels, over and above their relation to indecisiveness, previously established by Frost and Shows (1992).
(2) Maximization would be uniquely related to OCI-R scores, over and above its relation to depression and anxiety, previously established by Schwartz (2004).
(3) SPISI scores would partly account for the relation between OCI-R scores and maximization.

In addition, in Study 2 we predicted that:

(4) OCI-R scores would be related to intentions to search smartphone-related information extensively (comparing as many brands as possible, reading many reviews, spending many hours on the comparison etc.).
(5) SPISI scores would account, at least partly, for the relation between OCI-R scores and intentions to search smartphone-related information.

## Results

Means, standard deviations and Cronbach’s α of all measures used in Study 1 and in Study 2 are displayed in Supplementary Table 1. Consistent with our prediction, OC tendencies as measured by the OCI-R were positively associated with maximization in both studies (Study 1: *r*(199) = 0.54, *p* < 0.001, 95% CI [0.43,0.63], Study 2: *r*(238) = 0.29, *p* < 0.001, 95% CI [0.17,0.40]). Moreover, significant positive correlations with maximization emerged for each subscale of the OCI-R (see Supplementary Table 2).

Replicating the findings of Frost and Shows (1992), the OCI-R correlated positively with indecisiveness (Study 1: *r*(199) = 0.47, *p* < 0.001, 95% CI [0.35,0.57], Study 2: *r*(238) = 0.37, *p* < 0.001, 95% CI [0.25,0.47].) Significant positive correlations with indecisiveness emerged for each subscale of the OCI-R, except for *ordering* [Study 1: *r*(199) = 0.08, *p* = 0.248, Study 2: *r*(238) = 0.12, *p* = 0.057].

Maximization and indecisiveness were positively but not highly correlated with each other (Study 1: *r*(199) = 0.25, *p* < 0.001, 95% CI [0.12,0.38], Study 2: *r*(238) = 0.19, *p* < 0.001, 95% CI [0.06,0.31]), supporting our notion that the two scales gauge different aspects of difficulty in decision making. In addition, replicating previous findings (e.g., Schwartz, 2004), maximization was positively related to both depression (Study 1: *r*(199) = 0.32, *p* < 0.001, 95% CI [0.19,0.44], Study 2: *r*(238) = 0.25, *p* < 0.001, 95% CI [0.13,0.36]) and anxiety (Study 1: *r*(199) = 0.40, *p* < 0.001, 95% CI [0.28,0.51], Study 2: *r*(238) = 0.26, *p* < 0.001, 95% CI [0.14,0.37])^3^.

### Are OC Tendencies Positively Related to Maximization, Over and Above Their Relation to Indecisiveness?

Is the relation between OC tendencies and maximization due to the positive relation of each of these constructs with indecisiveness? We predicted that this would not be the case. Rather, we predicted that maximization would be related to OC tendencies even when indecisiveness is controlled for. To test this prediction, we conducted two two-stage hierarchical regression analyses with OC tendencies as the dependent variable. In the first analysis, indecisiveness was entered in the first stage and maximization was entered in the second stage. In the second analysis, maximization was entered in the first stage and indecisiveness was entered second.

In both studies, the first analysis (see **Table 1** below) revealed that at Stage 1 of the regression, indecisiveness contributed significantly to the regression model [Study 1: *F*(1,199) = 56.91, *p* < 0.001, *R^2^* = 22.2%, Study 2: *F*(1,238) = 36.95, *p* < 0.001, *R^2^* = 13.4%] and that at Stage 2 of the regression, maximization contributed significantly to the regression model as well [Study 1: *F*(1,198) = 64.66, *p* < 0.001, *ΔR^2^* = 19.1%, Study 2: *F*(1,237) = 14.46, *p* < 0.001, *ΔR^2^* = 5.0%].

**Table 1:** Summary of hierarchical regression analysis predicting OC tendencies from indecisiveness at Stage 1 and maximization at Stage 2.

Variable	Model 1			Model 2		
	*β*	*t*	*p*	*β*	*t*	*p*
Study 1 (*N* = 201)		
Indecisiveness	0.47	7.54	<0.001	0.36	6.33	<0.001
Maximization				0.45	8.04	<0.001
Study 2 (*N* = 240)		
Indecisiveness	0.37	6.08	<0.001	0.32	5.39	<0.001
Maximization				0.23	3.80	<0.001

The second analysis (see **Table 2**) revealed that at Stage 1 of the regression, maximization contributed significantly to the regression model [Study 1: *F*(1,199) = 83.34, *p* < 0.001, *R^2^* = 29.5%, Study 2: *F*(1,238) = 21.91, *p* < 0.001, *R^2^* = 8.4%]. Additionally, introducing indecisiveness in Stage 2 of the analysis contributed significantly to the regression model [Study 1: *F*(1,198) = 40.07, *p* < 0.001, *ΔR^2^* = 11.9%, Study 2: *F*(1,237) = 29.02, *p* < 0.001, *ΔR^2^* = 10.0%]. Together, these analyses suggest that indecisiveness and maximization gauge different aspects of decision making difficulties, both of which are uniquely related to OC tendencies.

**Table 2:** Summary of hierarchical regression analysis predicting OC tendencies from maximization at Stage 1 and indecisiveness at Stage 2.

Variable	Model 1			Model 2		
	*β*	*t*	*p*	*β*	*t*	*p*
Study 1 (*N* = 201)		
Maximization	0.54	9.13	<0.001	0.45	8.04	<0.001
Indecisiveness				0.36	6.33	<0.001
Study 2 (*N* = 240)		
Maximization	0.29	4.68	<0.001	0.23	3.80	<0.001
Indecisiveness				0.32	5.39	<0.001

### Is Maximization Related to OC Tendencies Over and Above Its Relation to Depression and Anxiety?

Is it possible that OC tendencies are related to maximization because both are related to depression and anxiety? We predicted that this would not be the case, but rather that the correlation between maximization and OC tendencies would remain even after depression and anxiety are controlled for. To examine this prediction, we conducted two two-stage multiple hierarchical regression analyses with maximization as the dependent variable.

In the first regression (see **Table 3**), depression and anxiety (as measured by the DASS) were entered at Stage 1 of the regression and OC tendencies (as measured by the OCI-R) were entered at Stage 2 of the analysis. The analysis revealed that at Stage 1, depression and anxiety contributed significantly to the regression model [Study 1: *F*(2,198) = 18.50, *p* < 0.001, *R^2^* = 15.7%, Study 2: *F*(2,237) = 9.22, *p* < 0.001, *R^2^* = 7.2%]. Adding OC tendencies at Stage 2 of the analysis contributed significantly to the regression model [Study 1: *F*(1,197) = 41.08, *p* < 0.001, *ΔR^2^* = 14.5%, Study 2: *F*(1,236) = 6.70, *p* = 0.010, *ΔR^2^* = 2.6%].

**Table 3:** Summary of hierarchical regression analysis predicting maximization from depression and anxiety at Stage 1 and OC tendencies at Stage 2.

Variable	Model 1			Model 2		
	*β*	*t*	*p*	*β*	*t*	*p*
Study 1 (*N* = 201)		
Depression	0.03	0.32	0.748	-0.05	-0.55	0.58
Anxiety	0.07	3.49	0.001	0.14	1.38	0.17
OCI-R				0.49	6.41	<0.001
Study 2 (*N* = 240)		
Depression	0.09	0.83	0.406	0.04	0.36	0.720
Anxiety	0.18	1.62	0.107	0.11	0.95	0.344
OCI-R				0.20	2.59	0.010

In the second regression analysis (see **Table 4**), OCI-R was entered at Stage 1 of the analysis and depression and anxiety levels were entered at Stage 2. At Stage 1, OC tendencies contributed significantly to the regression model [Study 1: *F*(1,199) = 83.34, *p* < 0.001, *R^2^* = 29.5%, Study 2: *F*(1,238) = 21.91, *p* < 0.001, *R^2^* = 8.4%]. Introducing depression and anxiety in Stage 2 did not significantly add to the explained variance [Study 1: *F*(2,197) = 1.09, *p* = 0.339, *ΔR^2^* = 0.8%, Study 2: *F*(2,236) = 1.77, *p* = 0.173, *ΔR^2^* = 1.4%]. These results support the notion that the relationship between maximization and OC tendencies is not mediated by depression/anxiety and in fact suggest that the relationship between maximization and depression/anxiety may be due to the tendency of depression and anxiety to co-occur with OC tendencies (as we further elaborate in the Section “Discussion”).

**Table 4:** Summary of hierarchical regression analysis predicting maximization from OC tendencies at Stage 1 and depression and anxiety at Stage 2.

Variable	Model 1			Model 2		
	*β*	*t*	*p*	*β*	*t*	*p*
Study 1 (*N* = 201)		
OCI-R	0.54	9.13	<0.001	0.49	6.41	<0.001
Depression				-0.05	-0.55	0.581
Anxiety				0.14	1.38	0.170
Study 2 (*N* = 240)		
OCI-R	0.29	4.68	<0.001	0.20	2.59	0.010
Depression				0.04	0.36	0.720
Anxiety				0.11	0.95	0.344

### Does the Tendency to Seek Proxies for Internal States Account for the Relation Between OC Tendencies and Maximization?

We theorized that OC tendencies manifest in a maximizing decision making style because such tendencies are associated with deficient access to internal states and with seeking proxies for those states. We thus hypothesized that scores on the SPISI, which gauges these constructs, would account, at least partly, for the relation between OC tendencies and maximization. To examine this prediction, we conducted two two-stage hierarchical multiple regression analyses with maximization as the dependent variable.

In the first regression (see **Table 5**), OC tendencies (as measured by the OCI-R) were entered at Stage 1 of the analysis and seeking proxies for internal states (as measured by the SPISI) were entered at Stage 2 of it. The analysis revealed that at Stage 1, OC tendencies contributed significantly to the regression model [Study 1: *F*(1,199) = 83.34, *p* < 0.001, *R^2^* = 29.5%, Study 2: *F*(1,238) = 21.91, *p* < 0.001, *R^2^* = 8.4%]. Importantly, adding Seeking proxies for internal states also contributed significantly to the model [Study 1: *F*(1,198) = 31.78, *p* < 0.001, *ΔR^2^* = 9.7%, Study 2: *F*(1,237) = 10.22, *p* = 0.002, *ΔR^2^* = 3.8%].

**Table 5:** Summary of hierarchical regression analysis predicting maximization from OC tendencies at Stage 1 and seeking proxies for internal states at Stage 2.

Variable	Model 1			Model 2		
	*β*	*t*	*p*	*β*	*t*	*p*
Study 1 (*N* = 201)		
OCI-R	0.54	9.13	>0.001	0.24	3.05	0.003
SPISI				0.44	5.64	>0.001
Study 2 (*N* = 240)		
OCI-R	0.29	4.68	<0.001	0.13	1.61	0.108
SPISI				0.25	3.20	0.002

The second regression analysis with maximization as the dependent measure introduced seeking proxies for internal states at Stage 1 of the analysis and OC tendencies at Stage 2 (see **Table 6**). The analysis revealed that at Stage 1, seeking proxies for internal states contributed significantly to the regression model [Study 1: *F*(1,199) = 113.98, *p* < 0.001, *R^2^* = 36.4%, Study 2: *F*(1,238) = 30.17, *p* < 0.001, *R^2^* = 11.3%]. OC tendencies contributed significantly to the model in Study 1 [*F*(1,198) = 9.28, *p* = 0.003, *ΔR^2^* = 2.8%], but not in Study 2 [*F*(1,237) = 2.60, *p* = 0.108, *ΔR^2^* = 1.0%]. These findings suggest that seeking proxies for internal states may partly account for the relationship between OC tendencies and maximization.

**Table 6:** Summary of hierarchical regression analysis predicting maximization from seeking proxies for internal states at Stage 1 and OC tendencies at Stage 2.

Variable	Model 1			Model 2		
	*β*	*t*	*p*	*β*	*t*	*p*
Study 1 (*N* = 201)		
SPISI	0.60	10.68	<0.001	0.44	5.64	<0.001
OCI-R				0.24	3.05	0.003
Study 2 (*N* = 240)		
SPISI	0.33	5.49	<0.001	0.25	3.20	0.002
OCI-R				0.13	1.61	0.108

### The Smartphone Decision Index (Study 2)

We standardized participants’ ratings on each of the nine questions and then calculated a “smartphone decision index” for each participant by averaging all nine questions (Cronbach’s α = 0 .76). The smartphone decision index reflects participants’ level of reliance on external cues in the course of making the decision, as well as their tendency to maximize by examining many alternatives.

In accordance with our hypotheses, we found positive correlations between the smartphone decision index and both the OCI-R, *r*(239) = 0.27, *p* < 0.001, 95% CI [0.15,0.38], and the SPISI, *r*(239) = 0.20, *p* < 0.001, 95% CI [0.08,0.32]. The smartphone decision index was also positively associated with depression, *r*(239) = 0.19, *p* = 0.003, 95% CI [0.06,0.31], and anxiety, *r*(239) = 0.14, *p* = 0.025, 95% CI [0.01,0.26] (for all correlations see Supplementary Table 3).

We followed the same analytic strategy as above to test the prediction that OC tendencies would be uniquely related to the smartphone decision index, over and above its relation to depression and anxiety. We also hypothesized that the relationship between the smartphone decision index and OC tendencies would be accounted for, at least partly, by the tendency to seek proxies for internal states as measured by the SPISI.

### Is the Smartphone Decision Index Positively Related to OC Tendencies, Over and Above Its Relation to Depression and Anxiety?

We predicted that the correlation between the smartphone decision index and OC tendencies would remain even after depression and anxiety would be controlled for. To examine this hypothesis, we conducted two-stage hierarchical multiple regressions with the smartphone decision index as the dependent variable. In the first analysis (see **Table 7** below), depression and anxiety (as measured by the DASS) were entered at Stage 1 of the regression and OC tendencies (as measured by the OCI-R) were entered at Stage 2. The analysis revealed that at Stage 1, depression and anxiety contributed significantly to the regression model, *F*(2,237) = 4.42, *p* = 0.013, *R^2^* = 3.6%. Adding OC tendencies in Stage 2 contributed significantly to the model, *F*(1,236) = 5.02, *p* = 0.026, *ΔR^2^* = 2.0%.

**Table 7:** Summary of hierarchical regression analysis predicting the smartphone decision index from depression and anxiety at Stage 1 and OC tendencies at Stage 2 (Study 2, *N* = 240).

Variable	Model 1			Model 2		
	*β*	*t*	*p*	*β*	*t*	*p*
Depression	0.22	1.93	0.055	0.18	1.50	0.134
Anxiety	-0.04	-0.37	0.713	-0.12	-0.91	0.363
OCI-R				0.18	2.24	0.026

In the second multiple regression analysis (see **Table 8**), OC tendencies were entered at Stage 1 of the analysis and depression and anxiety at Stage 2 of it. The analysis revealed that at Stage 1, OC tendencies contributed significantly to the regression model, *F*(1,238) = 11.61, *p* = 0.001, *R^2^* = 4.7%. Introducing depression and anxiety in Stage 2 of the regression did not significantly contribute to the model, *F*(2,236) = 1.19, *p* = 0.307, *ΔR^2^* = 0.9%. These findings suggest that the relationship between the smartphone decision index and OC tendencies cannot be explained by depression/anxiety. However, the relationship between the smartphone decision index and depression/anxiety seems to reflect the tendency of depressed and anxious people to have also OC tendencies.

**Table 8:** Summary of hierarchical regression analysis predicting the smartphone decision index from OC tendencies at Stage 1 and depression and anxiety at Stage 2 (Study 2, *N* = 240).

Variable	Model 1			Model 2		
	*β*	*t*	*p*	*β*	*t*	*β*
OCI-R	0.22	3.41	0.001	0.18	2.24	0.026
Depression				0.18	1.50	0.134
Anxiety				-0.11	-0.91	0.363

### Does the Tendency to Seek Proxies for Internal States Account for the Relation Between OC Tendencies and the Smartphone Decision Index?

We hypothesized that SPISI scores would account, at least to a certain extent, for the relation between OC tendencies and the smartphone decision index. To examine this prediction we conducted two two-stage hierarchical regression analyses with the smartphone decision index as the dependent variable. In the first analysis (see **Table 9**), OC tendencies (as measured by the OCI-R) were entered at Stage 1 of the analysis and seeking proxies for internal states (as measured by the SPISI) was entered at the second stage of the analysis. The analysis revealed that at Stage 1, OC tendencies contributed significantly to the regression model, *F*(1,238) = 11.61, *p* = 0.001, *R^2^* = 4.7%. Yet at Stage 2, seeking proxies to internal states (as measured by the SPISI) did not contribute significantly to the model, *F*(1,237) = 1.75, *p* = 0.187, *ΔR^2^* = 0.7%.

**Table 9:** Summary of hierarchical regression analysis predicting the smartphone decision index from OC tendencies at Stage 1 and seeking proxies for internal states at Stage 2 (Study 2, *N* = 240).

Variable	Model 1			Model 2		
	*β*	*t*	*p*	*β*	*t*	*p*
OCI-R	0.22	3.41	0.001	0.15	1.77	0.078
SPISI				0.11	1.32	0.187

In the second hierarchical multiple regression (see **Table 10**), seeking proxies for internal states was entered at Stage 1 and OC tendencies were entered at Stage 2. The analysis revealed that at Stage 1, seeking proxies for internal states contributed significantly to the regression model, *F*(1,238) = 10.18, *p* = 0.002, *R^2^* = 4.1%. Introducing OC tendencies in Stage 2 of the regression did not contribute significantly to the model, *F*(1,237) = 3.13, *p* = 0.078, *ΔR^2^* = 1.3%. This pattern of results suggests that OC tendencies and seeking proxies for internal states explain the same variance of the smartphone decision index. In other words, it is the variance that is common between the SPISI and the OCI-R that co-varies with the smartphone decision index.

**Table 10:** Summary of hierarchical regression analysis predicting the smartphone decision index from seeking proxies for internal states at Stage 1 and OC tendencies at Stage 2 (Study 2, *N* = 240).

Variable	Model 1			Model 2		
	*β*	*t*	*p*	*β*	*t*	*p*
SPISI	0.20	3.19	0.002	0.11	1.32	0.187
OCI-R				0.15	1.77	0.078

## Discussion

In two studies, we found that increased OC tendencies are associated with a tendency to maximize in decision making, that is, to continue seeking for better alternatives even after one has reached a satisfactory one (Schwartz, 2000; Schwartz et al., 2002). Importantly, this relation was not accounted for by depression and/or anxiety, and could not be explained by mere indecisiveness (Frost and Shows, 1992). A similar pattern of results emerged when we looked at maximization in a more concrete context of choosing a new smartphone (Study 2).

The association between OC tendencies and maximization is consistent with the SPIS model of OCD (Liberman and Dar, 2009; Lazarov et al., 2010), according to which this disorder is characterized by deficient access to internal states and as a consequence, a tendency to rely excessively on external proxies. Applied to decision making, the SPIS model suggests that OC individuals would have difficulty accessing a sense of satisfaction with the search one performed up to a certain point, as well with the chosen alternative. As a result, they would continue searching for a better alternative and seek external decision aids in the form of other people’s reviews and ratings and any other objective criteria. Consistent with this theorizing, in both studies we found that scores on an inventory that gauged the extent of relying on proxies for internal states (the Seeking Proxies for Internal States Inventory; Liberman and Dar, unpublished) accounted for some of the covariance between OC tendencies and maximization.

The results of this study replicate and extend the findings of Frost and Shows (1992), who showed that indecisiveness positively correlated with OC tendencies. We postulated, however, that maximization is a unique decision making difficulty, one that is not sufficiently reflected in the construct of indecisiveness as measured by Frost and Show’s scale. Supporting this postulation, we found only moderate correlations between indecisiveness and maximization. Moreover, OC tendencies were uniquely related to each of these two constructs (indecisiveness and maximization), suggesting that each of them captures different aspects of the difficulty that people with high OC tendencies have with making decisions.

Numerous theoretical frameworks and empirical studies have posited and demonstrated a relation between OC tendencies and perfectionism (e.g., McFall and Wollersheim, 1979; Mallinger, 1984; Rasmussen and Eisen, 1989; Janet, 1903; Frost et al., 1994; Frost and Steketee, 1997; Coles et al., 2003). Perfectionism involves setting excessively high personal standards of performance, accompanied by a tendency to evaluate one’s own behavior in an extremely critical manner (Frost et al., 1990). In thinking about perfectionism and its relation to OCD in terms of the SPIS model, we can distinguish between two different types of perfectionists: the first has a strong internal sense of what s/he wants and would clearly recognize the perfect option once s/he finds it. For example, an artist might look for the perfect composition with a strong sense of what feels right and what feels wrong. The second perfectionist cannot experience satisfaction either with the search s/he has performed or with what s/he has chosen. Knowing that better options could, in principle, exist, s/he is bound to continue searching for that illusive perfect option (for a related discussion of the relationship between perfectionism and maximization see Schwartz, 2004). Our studies suggest that OC tendencies would be related to the second type of perfectionism but not to first one, a prediction that can be tested in future studies.

As noted above, Schwartz et al. (2002) found that maximizers were particularly prone to depression. Though highly speculatively, our findings propose that the relationship between maximization and depression may be mediated by OC tendencies. Previous studies have repeatedly found high comorbidity between OCD and depression (and anxiety, see Bartz and Hollander, 2006). Our finding that levels of depression and anxiety did not contribute substantially to the relationship between OC tendencies and maximization might imply that the tendency to maximize is related to depression and anxiety only when accompanied by OC tendencies.

The current studies were based on a non-clinical population. The generalization of the findings to OCD requires a replication with a sample of OCD patients, preferably compared to patients diagnosed with other psychopathologies (e.g., depression and anxiety disorders). Although we controlled for depression and anxiety levels in our analyses, the comparison between patients suffering from OCD and patients suffering from depression and anxiety would strengthen the findings and indicate that the tendency to maximize is indeed specific to OCD and cannot be accounted for by comorbidity with other disorders. Furthermore, subsequent studies should, in addition to self-report scales, use various decision making behavioral paradigms. It would also be interesting to conduct online non-reactive content analyses in the field of applied decision making (e.g., consumer behaviors), in order to investigate OC individuals’ internet surfing patterns. Such analyses could include data about the time spent in websites offering comparative reviews and other surfers’ ratings of products, for instance.

The futility of maximizing is glaring and is relatively easy to explain, which makes the problem of a maximization strategy a good entry point for therapy with OC clients. Moreover, many individuals with OCD seek psychological treatment because they are tormented by obsessions related to important life decision, such as what to study at the University, whether or not to leave a job or marry one’s partner. The framework we propose here suggests that in such cases, it would be useful to examine with the client whether any doubts s/he experiences are in fact side effects of maximization, and to walk the client through the problems inherent in such maximization. It would also be important to help the client connect to his/her internal state of satisfaction with and liking of the alternative s/he considers rejecting in search for a better one. How does it feel to be with the partner whom you hesitate whether or not to marry? Do you feel excited by the new job? Are you happy to come back to your apartment at the end of the day? It is the answers to these questions, rather than the possibility that there might be something better out there, that should guide decisions. Potentially, therapists can encourage OC patients to gradually change their decision style by choosing alternatives which are “good enough,” rather than striving to reach “the best,” to minimize the time they spent comparing different alternatives and seeking objective criteria and to inhibit their excessive reliance on other people’s input.

To conclude, consistent with the SPIS model of OCD, OC tendencies were found to be associated with a maximization decision style. We suggest that this is the case because OC individuals cannot rely on their own sense of satisfaction with either the alternatives or the process of search. As a result, they are forced to continue searching and seek out objective criteria and other people’s decisions, opinions, and ratings. Identifying and explaining these decision making processes in OC individuals is an important step toward helping those individuals learn to identify and control their own internal states, to moderate their use of external proxies and eventually, to make decisions more efficiently.

## Data Availability

The raw data supporting the conclusions of this manuscript will be made available by the authors, without undue reservation, to any qualified researcher.

## Author Contributions

EO, RD, and NL conceived the presented research questions and planned the two studies accordingly. EO performed the statistical calculations with support from RD and NL. All three authors contributed to the interpretation of the results. EO took the lead in writing the manuscript. RD and NL provided critical feedback on the manuscript.

## Conflict of Interest Statement

The authors declare that the research was conducted in the absence of any commercial or financial relationships that could be construed as a potential conflict of interest.

## Footnotes

1 http://www.midgam.com

2 www.prolific.ac

3 Note that Study 2 used an online student sample and therefore, compared to Study 1, the mean age was lower (and its standard deviation smaller) and the mean years of formal education was higher. Because maximization was negatively related to age in Study 1 (*r* = -0.29), such that maximization characterized younger participants, maximization scores were relatively high (and the variance in maximization relatively low) in Study 2 which, as noted above, comprised younger participants. This could explain the smaller correlation between maximization and other variables (OC tendencies, seeking proxies for internal states etc.) in Study 2 compared to Study 1.

## Appendix 1

The questionnaires administrated in Study 1 and in Study 2.

OCI-R (Foa et al., 2002)

The following statements refer to experiences that many people have in their everyday lives.

Circle the number that best describes HOW MUCH that experience has DISTRESSED or BOTHERED you during the PAST MONTH. The numbers refer to the following verbal labels:

     0 = Not at all
     1 = A little
     2 = Moderately
     3 = A lot
     4 = Extremely

(1) I have saved up so many things that they get in the way.
(2) I check things more often than necessary.
(3) I get upset if objects are not arranged properly.
(4) I feel compelled to count while I am doing things.
(5) I find it difficult to touch an object when I know it has been touched by strangers or certain people.
(6) I find it difficult to control my own thoughts.
(7) I collect things I don’t need.
(8) I repeatedly check doors, windows, drawers, etc.
(9) I get upset if others change the way I have arranged things.
(10) I feel I have to repeat certain numbers.
(11) I sometimes have to wash or clean myself simply because I feel contaminated.
(12) I am upset by unpleasant thoughts that come into my mind against my will.
(13) I avoid throwing things away because I am afraid I might need them later.
(14) I repeatedly check gas and water taps and light switches after turning them off.
(15) I need things to be arranged in a particular order.
(16) I feel that there are good and bad numbers.
(17) I wash my hands more often and longer than necessary.
(18) I frequently get nasty thoughts and have difficulty in getting rid of them.

SPISI (Liberman and Dar, unpublished).

For each of the following items, please mark the extent to which the statement applies to you, using the following scale:

     1 = Not at all
     2 = A little
     3 = Moderately
     4 = Quite a bit
     5 = Very much

(1) I look for rules that would tell me what I’m supposed to do
(2) Sometimes I have to infer my feelings from my own actions
(3) To know how hungry I am, I consider what and when I’ve eaten today
(4) I turn to others to know if I acted right
(5) I find it difficult to form an opinion about a person without hearing other opinions
(6) I need clear evidence to be sure what others think about me
(7) I tend to consult others about daily decisions
(8) To know if I have understood what I’ve read, I check to see if I remember parts of it by heart
(9) When choosing, I prefer to use clear criteria rather than intuition
(10) I would prefer to use a formula to solve a math problem even if I think I know the answer
(11) Because I have difficulty deciding, I’ve developed fixed rules
(12) I know how close I am to someone by how often we interact
(13) I know if I’ve enjoyed my vacation based on how much I have done
(14) I choose what to wear based on pre-determined criteria
(15) I am only sure I understand what I’ve studied if I receive a good grade on the exam

DASS (Lovibond and Lovibond, 1995)

Please read each statement and circle a number 0, 1, 2, or 3 which indicates how much the statement applied to you over the past week. There are no right or wrong answers. Do not spend too much time on any statement. The rating scale is as follows:

     0 = Did not apply to me at all
     1 = Applied to me to some degree, or some of the time
     2 = Applied to me to a considerable degree, or a good part of time
     3 = Applied to me very much, or most of the time

(1) I was aware of dryness of my mouth
(2) I couldn’t seem to experience any positive feeling at all
(3) I experienced breathing difficulty (e.g., excessively rapid breathing, breathlessness in the absence of physical exertion)
(4) I just couldn’t seem to get going
(5) I had a feeling of shakiness (e.g., legs going to give way)
(6) I found myself in situations that made me so anxious I was most relieved when they ended
(7) I felt that I had nothing to look forward to
(8) I felt sad and depressed
(9) I had a feeling of faintness
(10) I felt that I had lost interest in just about everything
(11) I felt I wasn’t worth much as a person
(12) I perspired noticeably (e.g., hands sweaty) in the absence of high temperatures or physical exertion
(13) I felt scared without any good reason
(14) I felt that life wasn’t worthwhile

Indecisiveness Scale (Frost and Shows, 1992)

For each of the following items, please mark the extent to which the statement applies to you, using the following scale:

     1 = Completely disagree
     2 = Quite disagree
     3 = Neutral
     4 = Quite agree
     5 = Completely agree

(1) I try to put off making decisions
(2) I always know exactly what I want
(3) I find it easy to make decisions
(4) I have a hard time planning my free time
(5) I like to be in a position to make decisions
(6) Once I make a decision, I feel fairly confident that it is a good one
(7) When ordering from a menu, I usually find it difficult to decide what to get
(8) I usually make decisions quickly
(9) Once I make a decision, I stop worrying about it
(10) I become anxious when making a decision
(11) I often worry about making the wrong choice
(12) After I have chosen or decided something, I often believe I’ve made the wrong choice or decision
(13) I do not get assignments done on time because I cannot decide what to do first
(14) I have trouble completing assignments because I can’t prioritize: what is most important
(15) It seems that deciding on the most trivial thing takes me a long time

Items 2, 3, 5, 6, 8, and 9 are reverse scored.

Maximization Scale (Nenkov et al., 2008)

For each of the following items, please mark the extent to which the statement applies to you, using the following scale:

     1 = Completely disagree
     2 = Quite disagree
     3 = Neutral
     4 = Quite agree
     5 = Completely agree

(1) When I am in the car listening to the radio, I often check other stations to see if something better is playing, even if I am relatively satisfied with what I’m listening to
(2) No matter how satisfied I am with my job, it’s only right for me to be on the lookout for better opportunities
(3) I often find it difficult to shop for a gift for a friend
(4) Whenever I’m in a romantic relationship with a partner, I’m bothered by the thought that there may be a better fit out there for me
(5) No matter what I do, I have the highest standards for myself
(6) I never settle for second best

## Appendix 2

The smartphone decision index.

Imagine your smartphone broke down and you need to buy a new one today

(1) You can compare prices of different smartphone brands. How many brands would you like to compare? [just one brand∖ three brands∖ six brands∖ 10 brands∖ 30 brands]
(2) You can compare technical characteristics of different smartphone brands. How many brands would you like to compare? [just one brand∖ three brands∖ six brands∖ 10 brands∖ 30 brands]
(3) You can try using a smartphone for several hours. How many brands would you like to try? [just one brand∖ three brands∖ six brands∖ 10 brands∖ 30 brands]
(4) You can compare the camera characteristics of different smartphone brands. How many brands would you like to compare? [just one brand∖ three brands∖ six brands∖ 10 brands∖ 30 brands]
(5) You can read other users’ reviews of the specific brand you’re considering to buy. How many reviews would you like to read? [just one review∖ 2–3 reviews∖ 4–10 reviews∖ 11 reviews or more]
(6) You can receive information about the popularity of the specific brand you’re considering (compared to other brands) in different countries worldwide. Would you like to receive such information? [no∖ yes, but only in the United Kingdom∖ yes, but only in Europe∖ yes, I would be interested in its popularity worldwide]
(7) How much time, in hours, would you spend comparing the options and researching which smartphone to buy? [any number from 0]
(8) Once you’ve decided which smartphone to buy, how much time, in hours, would you spend finding the best deal? [any number from 0]
(9) If money were no object, it would be important to me to buy the best smartphone there is [from 1 = completely disagree, to 5 = completely agree]
