# Supplementary material for: Obsessive–Compulsive Tendencies Are Related to a Maximization Strategy in Making Decisions
Source: Front Psychol. 2018 May 22;9:778. doi: 10.3389/fpsyg.2018.00778 (PMC5972320; doi:10.3389/fpsyg.2018.00778)
Supplement: Supplementary file 1 [file Table_1.DOCX]

Table 1. Means, standard deviations and Cronbach’s α of the OCI-R, the SPISI, the depression and anxiety indices of the DASS, the maximization scale, the indecisiveness scale and the smartphone decision index.

| Measure | *M* | *SD* | | | Cronbach’s α | |
| --- | --- | --- | --- | --- | --- | --- |
| Study 1 (N = 201) |  |  | | |  | |
| OCI-R | 22.73 | 12.45 | | | 0.91 | |
| SPISI | 31.48 | 11.00 | | | 0.91 | |
| Depression | 4.07 | 4.64 | | | 0.90 | |
| Anxiety | 3.93 | 4.40 | | | 0.87 | |
| Maximization | 16.24 | 4.52 | | | 0.71 | |
| Indecisiveness | 36.80 | 9.52 | | | 0.86 | |
| Study 2 (N = 240) |  |  | | |  | |
| OCI-R | 19.48 | | 11.63 | 0.88 | |  |
| SPISI | 34.70 | | 11.03 | 0.89 | |  |
| Depression | 5.92 | | 4.57 | 0.85 | |  |
| Anxiety | 4.45 | | 3.97 | 0.83 | |  |
| Maximization | 18.82 | | 3.84 | 0.53^[[1]](#footnote-1)^ | |  |
| Indecisiveness | 45.52 | | 11.01 | 0.90 | |  |
| Smartphone decision index^[[2]](#footnote-2)^ | 0.01 | | 0.59 | 0.76 | |  |

1. Note that the means and standard deviations of all six items did not differ from each other, moreover, removing items does not improve Cronbach’s α. [↑](#footnote-ref-1)
2. The reported values are the standardized values (see the Results section in the main text). [↑](#footnote-ref-2)
