# Supplementary material for: Obsessive–Compulsive Tendencies Are Related to a Maximization Strategy in Making Decisions
Source: Front Psychol. 2018 May 22;9:778. doi: 10.3389/fpsyg.2018.00778 (PMC5972320; doi:10.3389/fpsyg.2018.00778)
Supplement: Supplementary file 2 [file Table_2.DOCX]

Table 2. Pearson correlations of indecisiveness and maximization with OCI-R (including subscales), SPISI, depression and anxiety.

| Measure | Indecisiveness | CI 95% | Maximization | CI 95% |
| --- | --- | --- | --- | --- |
| Study 1 (N = 201) |  |  |  |  |
| OCI-R obsessing | 0.50* | [.39, .60] | 0.40* | [.28, .51] |
| OCI-R ordering | 0.08 | - | 0.33* | [.20, .45] |
| OCI-R hoarding | 0.41* | [.29, .52] | 0.38* | [.25, .49] |
| OCI-R washing | 0.39* | [.27, .50] | 0.42* | [.30, .53] |
| OCI-R neutralizing | 0.43* | [.31, .54] | 0.43* | [.31, .54] |
| OCI-R checking | 0.38* | [.25, .49] | 0.52* | [.41, .61] |
| OCI-R total | 0.47* | [.35, .57] | 0.54* | [.43, .63] |
| SPISI | 0.47* | [.35, .57] | 0.60* | [.50, .68] |
| DASS depression | 0.54* | [.43, .63] | 0.32* | [.19, .44] |
| DASS anxiety | 0.42* | [.30, .53] | 0.40* | [.28, .51] |
| Maximization | 0.25* | [.12, .38] | - | - |
| Study 2 (N = 240) |  |  |  |  |
| OCI-R obsessing | 0.46* | [.35, .55] | 0.22* | [.10, .34] |
| OCI-R ordering | 0.12 | - | 0.24* | [.12, .36] |
| OCI-R hoarding | 0.33* | [.21, .44] | 0.13* | [.00, .25] |
| OCI-R washing | 0.14* | [.01, .26] | 0.22* | [.10, .34] |
| OCI-R neutralizing | 0.14* | [.01, .26] | 0.21* | [.09, .33] |
| OCI-R checking | 0.26* | [.14, .37] | 0.20* | [.08, .32] |
| OCI-R total | 0.37* | [.25, .47] | 0.29* | [.17, .40] |
| SPISI | 0.34* | [.22, .45] | 0.33* | [.21, .44] |
| DASS depression | 0.50* | [.40, .59] | 0.25* | [.13, .36] |
| DASS anxiety | 0.49* | [.39, .58] | 0.26* | [.14, .37] |
| Maximization | 0.19* | [.06, .31] | - | - |

*All marked (*) correlations are significant at the critical alpha of 0.001.*
