# Supplementary material for: Obsessive–Compulsive Tendencies Are Related to a Maximization Strategy in Making Decisions
Source: Front Psychol. 2018 May 22;9:778. doi: 10.3389/fpsyg.2018.00778 (PMC5972320; doi:10.3389/fpsyg.2018.00778)
Supplement: Supplementary file 3 [file Table_3.DOCX]

Table 3. Pearson correlations of the smartphone decision index with OCI-R, SPISI, depression, anxiety, indecisiveness and maximization (Study 2, N = 240).

| Measure | The smartphone decision index | CI 95% |
| --- | --- | --- |
| OCI-R total | 0.27* | [.15, .38] |
| SPISI | 0.20* | [.08, .32] |
| DASS depression | 0.19* | [.06, .31] |
| DASS anxiety | 0.14* | [.01, .26] |
| Indecisiveness | 0.11 | - |
| Maximization | 0.21* | [.09, .33] |

*All marked (*) correlations are significant at the critical alpha of 0.001.*
